# High cervical spinal cord stimulation in Parkinson’s disease with dopamine-resistant axial disabilities: a case with 2-year follow-up

**DOI:** 10.1007/s00415-023-11719-w

**Published:** 2023-04-17

**Authors:** Zhengyu Lin, Linbin Wang, Peng Huang, Yixin Pan, Yuyan Tan, Shengdi Chen, Dianyou Li

**Affiliations:** 1 Department of Neurosurgery, Ruijin Hospital Affiliated to Shanghai Jiao Tong University School of Medicine, 197 Ruijin 2nd Road, Shanghai, 200025 China; 2 Center for Functional Neurosurgery, Ruijin Hospital Affiliated to Shanghai Jiao Tong University School of Medicine, 197 Ruijin 2nd Road, Shanghai, 200025 China; 3 Institute of Science and Technology for Brain-Inspired Intelligence (ISTBI), Fudan University, 220 Handan Road, Shanghai, 200433 China; 4 Department of Neurology, Ruijin Hospital Affiliated to Shanghai Jiao Tong University School of Medicine, 197 Ruijin 2nd Road, Shanghai, 200025 China

Dear Sirs,

The management of disabling axial disabilities in advanced Parkinson’s disease (PD) is challenging since they are frequently insensitive to pharmacotherapy and deep brain stimulation (DBS). The epidural spinal cord stimulation (SCS) may be a surgical alternative for medically unresponsive axial symptoms in PD [1]. However, current evidence for the long-term effectiveness of SCS in treating pain-free idiopathic PD is limited. The thoracic SCS (tSCS) has been mainly reported in such PD population but with conflicting long-term results [2, 3]. In addition, the tSCS has barely any effect on upper axial functions. Here, we presented for the first time the long-term outcome of high cervical SCS (h-cSCS) on medically refractory gait disturbances and speech in a pain-free PD case.

A 75-year-old male suffering from bradykinesia and severe axial disabilities was referred to our center. He was diagnosed with idiopathic PD since 2014 and the initial levodopa replacement therapy was effective. Since 2019, the patient gradually developed postural instability, freezing of gait, and dysarthria, along with a considerable decline of levodopa responsiveness. The preoperative levodopa equivalent daily dose (LEDD) was 550 mg/day at admission [madopar (0.25 g/pill): 0.75–0.75–0.75 pill; selegiline oral: 5–5–0 mg]. A routine single-dose suprathreshold levodopa challenge test [4] showed a levodopa unresponsive pattern (MDS UPDRS score from 47 to 41). This patient was, therefore, not eligible for subthalamic or pallidal DBS surgery according to our routine screening criteria [4]. Regarding the experimental pedunculopontine nucleus (PPN) DBS, a recent randomized trial also did not demonstrated its efficacy in treating dopamine-resistant gait and balance issues in PD patients [5]. His eligibility for h-cSCS trial was then evaluated and favored by our multidisciplinary team. He underwent h-cSCS lead implantation with a modified retrograde surgical lead insertion technique uneventfully (see Supplementary Material). The postoperative radiography confirmed a satisfactory lead placement (Fig. 1A, B). We chose contacts 0–1 + , 10–2 + , 14–6 + , 15–7 + to cover the bilateral upper and lower limbs. The pulse width was set to 300 μs and the frequency was initially set to 60 Hz and was gradually reduced to 35 Hz at 2-year follow-up. The stimulation amplitude was position-adaptative and was ranged from 1.3 to 3.3 V at 2-year follow-up (Fig. 1C). Paresthesia was perceived as constant but well tolerated.

**Fig. 1:**
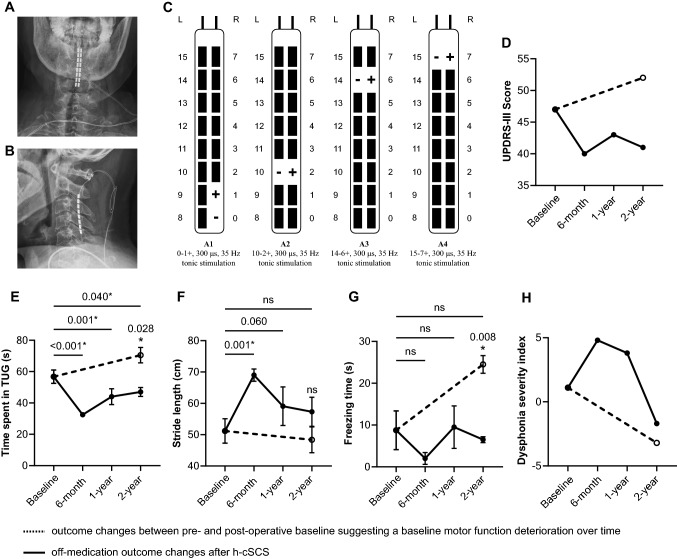
Stimulating parameters and outcome measures in the off-medication condition. **A**, **B** The postoperative antero-posterior (**A**) and lateral (**B**) cervical X-ray showed a satisfactory placement of the lead. **C** showed the optimal stimulating parameters at 2-year follow-up. The stimulating amplitude ranged from 1.3 to 3.3 V and was position-adaptive. Paresthesia was perceived as constant but was well tolerated. **D**–**H** Showed outcome measures including the unified Parkinson’s disease rating scale part III (**D**), time spent in Time-Up-and-Go test (**E**), stride length (**F**), freezing time (**G**), and dysphonia severity index (**H**). The black dot and the error bar indicated the mean and the standard deviation, respectively. The dashed line showed outcome changes between pre- and post-operative baseline suggesting the motor deterioration over time. For time spent in Time-Up-and-Go test, stride length, and freezing time, each was repeated measured for each medication/stimulation condition at each evaluation visit and compared using one-way ANOVA. The Dunnett’s t-test was used for multiple comparison correction for comparison with the preoperative baseline. The unpaired t-test was used for comparison between the off-medication/on-SCS condition and the postoperative baseline at 2-year follow-up. *p value < 0.05 was considered significant

All postoperative motor assessments were done in the off-medication condition. Overall motor function was assessed by the MDS Unified Parkinson Disease Rating Scale part III (UPDRS-III), gait performance by the 5-m Timed-Up-and-Go (TUG) test, and voice quality by the dysphonia severity index (DSI; lingWAVES, Atmos, Medizin Technik, Germany). The time spent in TUG, the average stride length, and the total freezing time were further obtained. To calculate the freezing time per session, each episode of freezing of gait was visually identified by an experienced rater after inspecting the videotape. This rater was blinded to the medication and stimulation conditions. The duration of each freezing of gait episode in one TUG test was then summed. The postoperative baseline (i.e., off-medication/off-SCS) of the MDS UPDRS-III score increased from 47 preoperatively to 52 at 2-year follow-up, suggesting a PD disease progression [6]. Despite that, the MDS UPDRS-III score was constantly improved at 2-year follow-up in the off-medication/on-SCS state (Fig. 1D). Similarly, compared to the preoperative baseline (56.8 ± 4.3), significant and sustained improvement in the time spent in TUG (in seconds) was observed at 6-months (32.5 ± 0.6, *p* < 0.001), 1-year (44.0 ± 5.1, *p* = 0.001), and 2-year (47.0 ± 2.8, *p* = 0.040) follow-up. The stride length (in centimeters) was significantly improved at 6-months follow-up (69.0 ± 1.9, *p* = 0.001) and insignificantly increased at both 1-year (59.1 ± 6.1, *p* = 0.060) and 2-year (57.3 ± 4.6, *p* = 0.348) review compared to the preoperative baseline (51.2 ± 3.9). Although the freezing time (in seconds) did not change from the preoperative baseline, it significantly ameliorated at 2-years follow-up compared to the postoperative baseline (6.5 ± 0.7 vs 24.5 ± 2.1, *p* = 0.008) (Fig. 1E–G, Video 1). In addition, the improvement in DSI score change was observed at 6-months (4.8) and 1-year (3.8) follow-up compared to the preoperative baseline (1.1), and was clinically meaningful in the long-term compared to the postoperative baseline (2-year: − 1.7 vs the postoperative baseline: − 3.2) (Fig. 1H). The LEDD at 2-year follow-up was 625 mg/days [madopar (0.25 g/pill): 0.75–0.75–0.75 pill; selegiline oral: 5–5–0 mg; pramipexole: 125–125–125 mg].

In fact, probably due to the following neuroanatomical reasons, most of the available studies have investigated tSCS rather than h-cSCS [3, 7, 8]. Firstly, the high thoracic spinal cord has a thinner diameter compared to the cervical level. In addition, ascending fibers from lower limbs course medially and occupy a deeper position in the dorsal columns at the thoracic level. Thus, tSCS may recruit a wider range of ascending fibers from lower limbs with similar stimulating thresholds and less undesirable paresthesia [1]. However, compared to tSCS, h-cSCS may have potential advantages, i.e., the putative beneficial modulating effects on cerebral blood flow [9] and upper appendicular and axial functions. As shown in this case, both upper and lower axial functionalities (i.e., DSI score and TUG time, respectively) were meaningfully improved in the long-term compared to the postoperative baseline. Given that PD patients with predominant gait disturbances often suffer from upper-axial disabilities, h-cSCS might be a more appropriate approach for pain-free PD population with refractory axial symptoms to some extent.

Evidence for h-cSCS in treating pain-free PD is scarce. To the best of our knowledge, this case firstly demonstrated the long-term motor benefits of h-cSCS in treating drug-resistant axial disabilities in pain-free PD patients. Thevathasan et al. reported negative results in two pain-free PD patients receiving percutaneous h-cSCS implantation [10]. However, they used percutaneous cylindrical leads (Medtronic models 3487a or 3898) of which the effective surface over the cervical spinal cord was latter shown to be insufficient compared to that used in the former rodent study [11]. Therefore, to reach a greater coverage on the cervical dorsal column in case of h-cSCS for pain-free PD, the surgical paddle lead, which was used in our case, should be a more rational choice. However, our previous work failed to observe statistically significant short-term therapeutic effects of h-cSCS using surgical paddle leads on parkinsonian gait disturbances. This may be probably due to the disease heterogeneity that pain-free PD and Parkinson variant of multiple system atrophy cases were pooled together for analysis in that study [12].

To conclude, h-cSCS showed therapeutic potential in improving drug-resistant axial disabilities in pain-free PD patients in the long-term. Further large-sample investigations are warranted.

## Supplementary Information

Below is the link to the electronic supplementary material.

Supplementary file1 (DOCX 413 KB)

Supplementary file2 (MP4 235542 KB)

## Data Availability

The data that support the findings of this study are available from the corresponding author upon reasonable request.
