# Supplementary material for: High cervical spinal cord stimulation in Parkinson’s disease with dopamine-resistant axial disabilities: a case with 2-year follow-up
Source: J Neurol. 2023 Apr 17;270(7):3650–3. doi: 10.1007/s00415-023-11719-w (PMC10267003; doi:10.1007/s00415-023-11719-w)
Supplement: Supplementary file 1 — Supplementary file1 (DOCX 413 KB) [file 415_2023_11719_MOESM1_ESM.docx]

**Supplementary Method**

*Surgical procedure*

We adopted a modified retrograde surgical electrode insertion technique for high cervical spinal cord stimulation (h-cSCS) surgery to avoid intraoperative X-ray guidance and radiation. The surgery is performed under general anesthesia. A single dose rocuronium is used for intubation induction without additional administration during the surgery to avoid false negative results during the intraoperative testing. The patient is in a prone position with head stabilized by the Mayfield frame, and with a roll placed under shoulders (Supplementary Figure 1A and 1B). A midline incision is made from the inion to C4 spinous process, and an exposure is performed from the posterior arch of C1 to spinous process of C3. Spinous processes of C2 and C3 should be removed, followed by two median laminotomies, measuring approximately 9 mm in width and 8 mm in height perpendicular to the spinal cord axis, at the level of the upper portion of the C2 and C3 lamina, respectively (Supplementary Figure 1C). The passage is then verified by the passing elevator (Supplementary Figure 1D), and the surgical paddle lead (2*8 contacts, model 39286, Medtronic, Minneapolis, MN) is placed downward from the C2 laminotomy window along the midline of the dura at C2 to C4. The electrode orientation can be directly visualized and easily adjusted from the C3 laminotomy window (Supplementary Figure 1E). The lead is then temporarily connected to the external stimulator (model 355531, Medtronic, Minneapolis, MN) for intraoperative testing after antagonizing the myorelaxant (sugammadex). The testing consists of examining the impedance of each contact and motor responses to a consecutive unilateral longitudinal bipolar setting of contacts from distal to proximal end to ensure a physiologically non-lateralized placement of the lead. Typically, we test three sets of stimulation parameters: 0-1+ for right-sided and 8-9+ for left-sided distal end, 3-4+ for right-sided and 11-12+ for left-sided middle part, and 6-7+ for right-sided and 14-15+ for left-sided proximal end. In general, the voltage, pulse width, and frequency are set to 3.0‒8.0 V, 60‒90 μs, 2 Hz, respectively. A stepwise increase in amplitude is applied to produce an ipsilateral muscle contraction at 2 Hz in the cervico-scapular region. After a satisfactory intraoperative testing, the lead was protected by silicone rubber and then anchored on the posterior tubercle of C1 with titanium plate to minimize lead migration risk due to cervical movement (Supplementary Figure 1F). We re-ensure lead placement after the fixation using the test pipeline mentioned above. Finally, the lead is connected to the portable stimulator by extension wire for two weeks during hospitalization. The postoperative test phase consists of clinical evaluations of axial symptoms (TUG test and UPDRS-III) in the on-medication/on-stimulation state during consecutive days with continuous stimulation. If the clinical response in the test phase is satisfactory with an improvement over 30% compared to the on-medication/off-stimulation state, the patient undergoes the second-stage surgery involving the subcutaneous implantation of the SCS neurostimulator (37714, Medtronic, Minneapolis, MN) in the subcalvicular region. The described technique has been performed in our center by a single neurosurgeon (Dr. Li) on several consecutive patients suffering from predominant drug-resistant parkinsonian gait disturbances^1^. One case with the longest (2-year) follow-up period was presented.

**References**

1. Wang L, Zhu R, Pan Y, et al. Effects of High Cervical Spinal Cord Stimulation on Gait Disturbance and Dysarthropneumophonia in Parkinson's Disease and Parkinson Variant of Multiple System Atrophy: A Case Series. Brain Sci 2022;12.


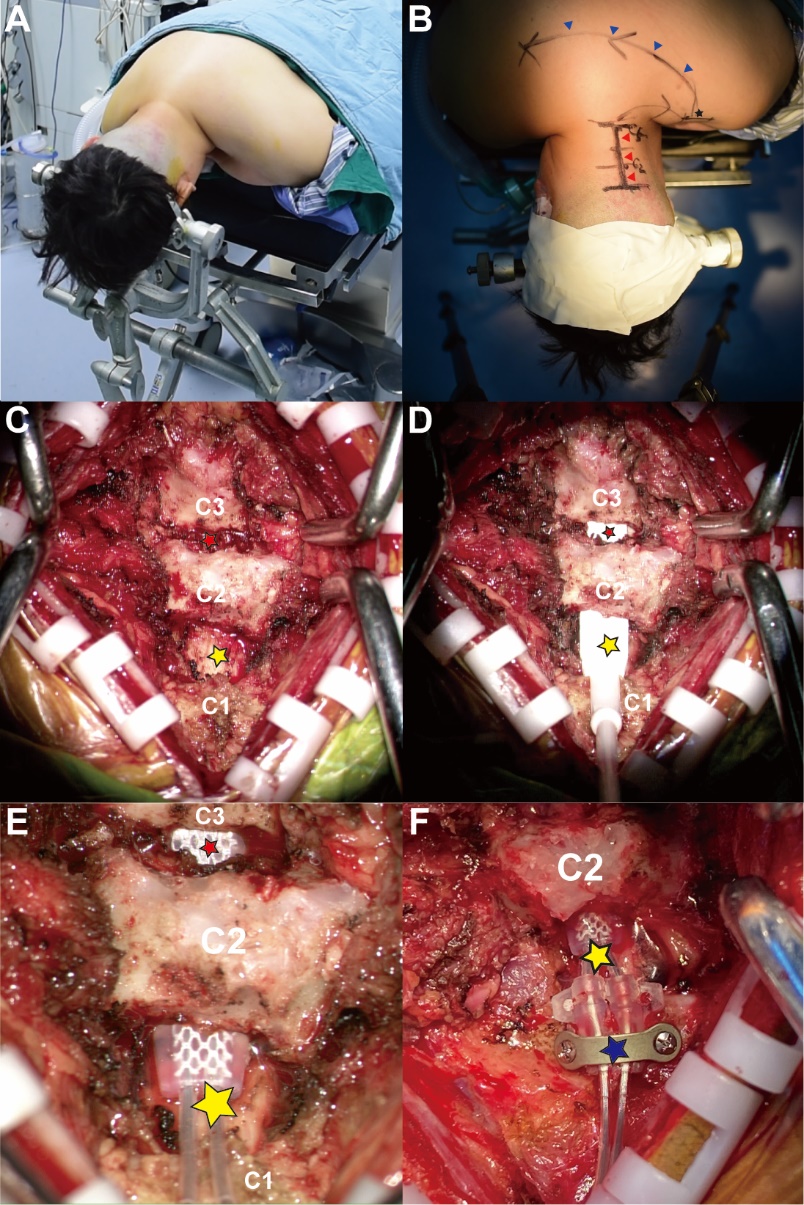


**Supplementary Figure 1. Illustration of the modified retrograde surgical lead insertion technique for high cervical spinal cord stimulation.** Installation (A) and incision (B): red triangles, blue triangles, and black asterisk indicates the incision line, subcutaneous tunnelization for temporary extension wire, and pocket for lead-extension junction, respectively. (C)-(F) illustrated the surgical procedure of this technique. The yellow, red, and blue asterisk indicates the C2 partial laminotomy, C3 partial laminotomy, and titanium plate on C1 posterior tubercle for lead fixation protected by the silicone rubber, respectively.
